# Case Report: Refractory *Mycoplasma pneumoniae* pneumonia complicated by pulmonary embolism and infarction in a child

**DOI:** 10.3389/fped.2025.1608233

**Published:** 2025-06-23

**Authors:** Jianqin Zhang, Zhe Zhang, Ziwei Zhu, Li Cheng, Yuxia Shan

**Affiliations:** ^1^ Pediatric Respiratory Department, Dalian Women and Children’s Medical Group, Dalian, Liaoning, China; ^2^ Department of Pharmacy, Dalian Women and Children’s Medical Group, Dalian, Liaoning, China

**Keywords:** refractory *Mycoplasma pneumoniae* pneumonia, severe *Mycoplasma pneumoniae* pneumonia, pulmonary embolism, children, infection

## Abstract

*Mycoplasma pneumoniae* (MP) is a significant pathogen of community-acquired pneumonia in children, typically following a benign course. However, some cases may progress to severe or refractory MP pneumonia (SMPP or RMPP) and lead to thromboembolic complications. This report describes a rare case of a 9-year-old boy with RMPP complicated by bilateral pulmonary embolism (PE) and pulmonary infarction. The patient initially presented with a fever and cough. Despite 24 days of prior treatment at another hospital, including macrolide, carbapenem, and tetracycline antibiotics and corticosteroids, he remained febrile with persistent wheezing when transferred to our institution. Through some laboratory findings and contrast-enhanced chest computed tomography, he fulfilled the diagnostic criteria for both SMPP and RMPP, accompanied by a PE with pulmonary infarction. A multidisciplinary therapeutic approach combining anti-infective agents (linezolid and moxifloxacin), anti-inflammatory therapy (methylprednisolone), and adjusted anticoagulation (low-molecular-weight heparin followed by rivaroxaban) led to rapid clinical improvement and normalization of inflammatory/coagulation markers. Complete resolution of the PE was further demonstrated by 3-month follow-up imaging. Residual focal necrosis in the right lower lobe was observed. This case highlights the potential for severe thromboembolic events in pediatric RMPP and underscores the importance of early recognition of imaging features (e.g., vascular filling defects and wedge-shaped infarcts) and integrated multidisciplinary management to optimize patient outcomes.

## 1 Introduction

*Mycoplasma pneumoniae* (MP) is one of the most common pathogens of community-acquired pneumonia (CAP) in children. An MP infection follows a largely benign course, while pneumonia is the most prominent clinical manifestation. *M. pneumoniae* pneumonia (MPP) accounts for up to 40% of CAP cases in children, with 18% of children requiring hospitalization (1). Children with MPP have been reported to have a high risk of blood coagulation and thrombosis (2–4). The mechanism of thrombosis caused by MPP is still unknown, but it is probably related to immune modulation (5). A pulmonary embolism (PE), the most common thromboembolism in patients with MPP, is a significant cause of residual atelectasis, organizing pneumonia, and pulmonary necrosis (6). Pediatric patients with a PE caused by MPP have rarely been reported. Herein, we report a case of a 9-year-old patient with refractory MPP (RMPP) who gradually developed a PE, and an etiological examination confirmed a *Haemophilus influenzae* superinfection. Following the administration of anticoagulation treatment, bronchoscopic interventional therapy, and systemic corticosteroid and antibiotic therapy, the patient demonstrated a favorable prognosis. This study aimed to contribute to the clinical understanding and management of such diseases by sharing practical diagnostic and therapeutic experiences.

## 2 Patient presentation

**Figure 1:**
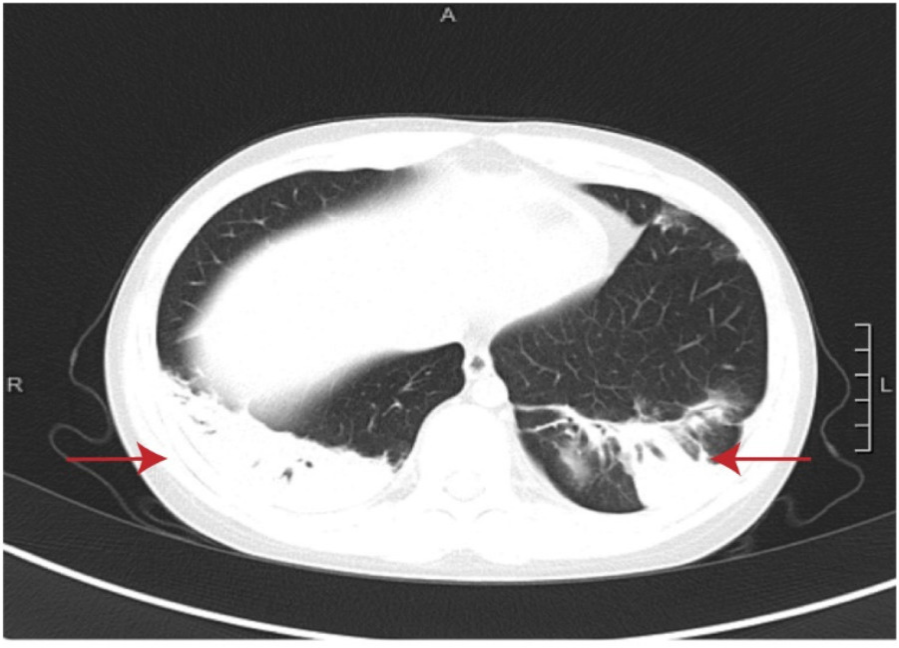
Chest CT in week 2 of the disease. The red arrows indicate wedge-shaped consolidations with pleural-based, hilar-pointing configurations.

**Figure 2:**
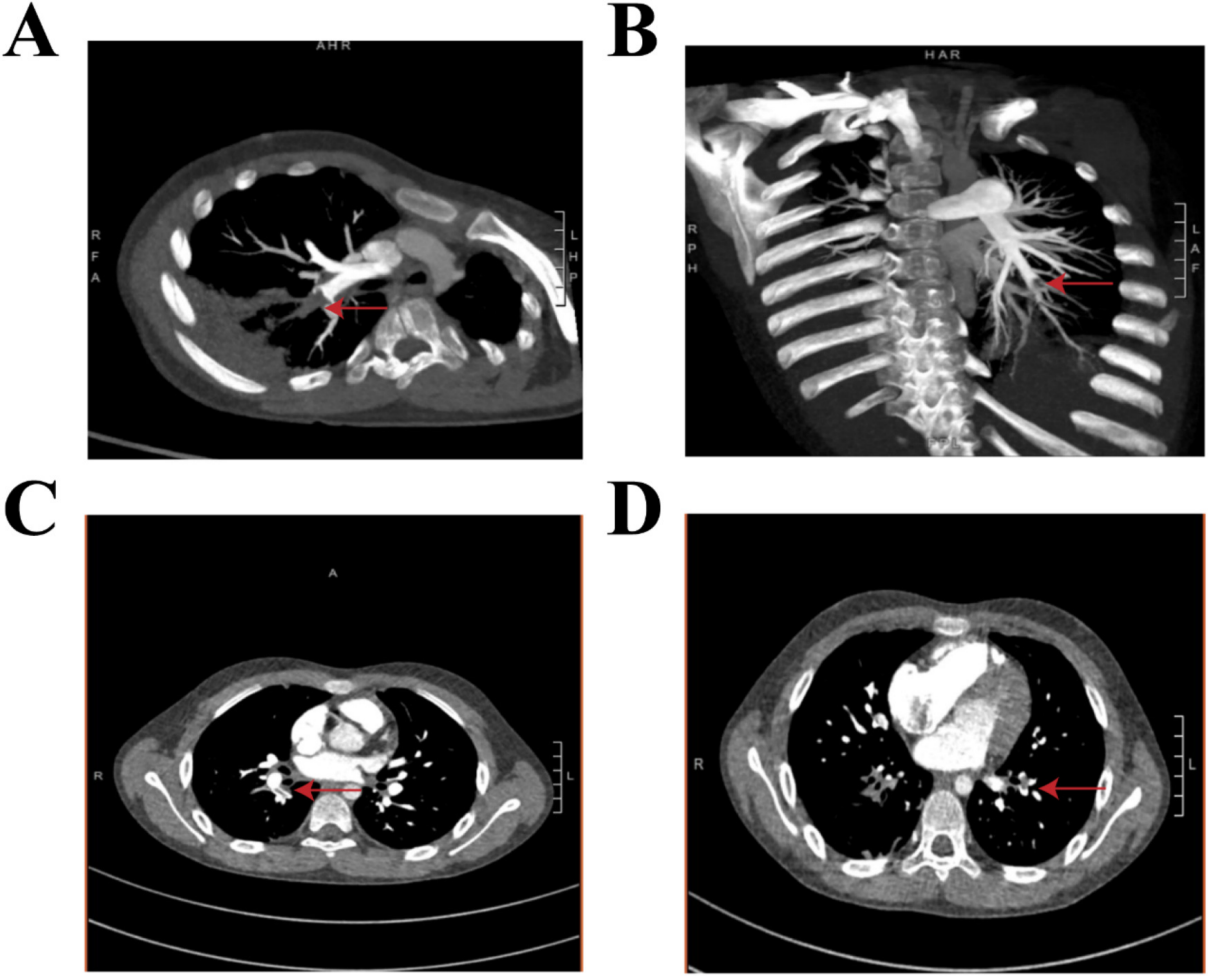
Contrast-enhanced chest CT obtained during the fourth week of illness demonstrates multiple pulmonary emboli in the bilateral lower lobe arteries. **(A**,**C)** The right lower lobe pulmonary artery shows filling defects involving the proximal basal segmental arteries (the red arrow) and their terminal branches. **(B**,**D)** The left lower lobe pulmonary artery shows occlusions at the origins of the basal segmental arteries (the red arrow), with distal propagation into branch vessels.

**Figure 3:**
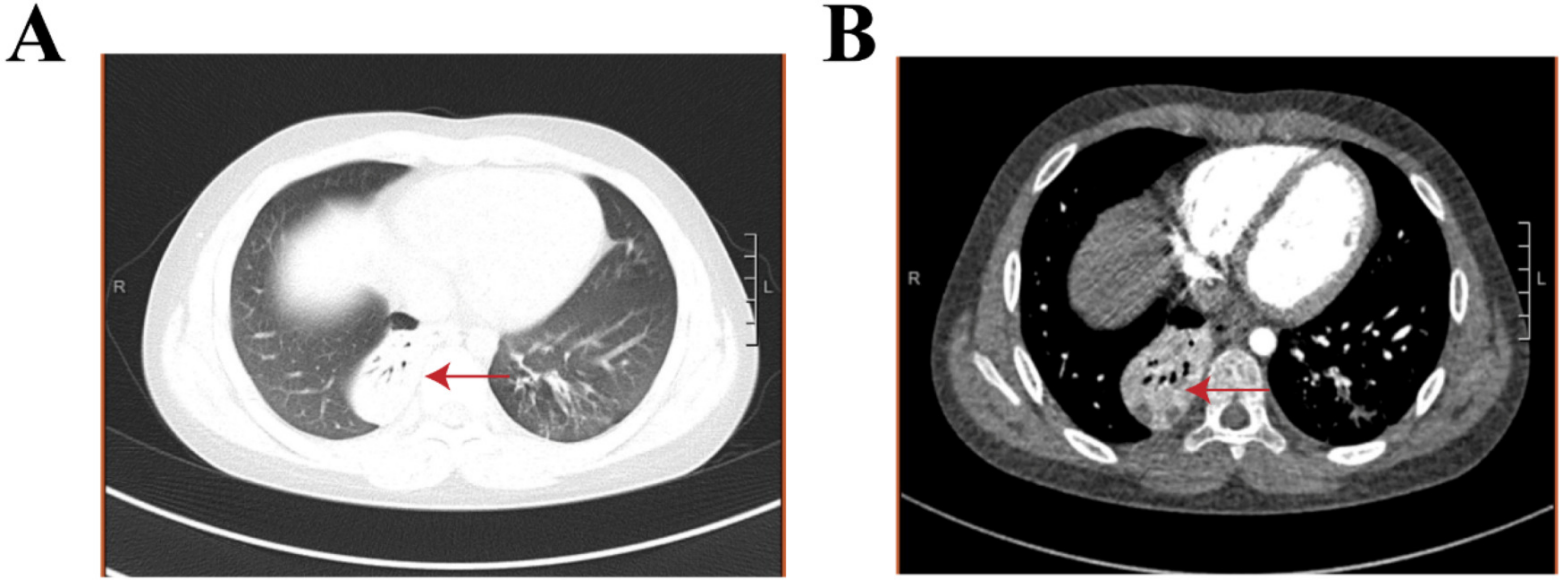
Chest contrast-enhanced CT with a three-dimensional reconstruction in month 4.5 of the disease. **(A, B)** The red arrows indicate right lower lobe pulmonary atelectasis with small areas of necrosis..

A 9-year-old boy with no significant family history, specifically nothing suggesting thromboembolic disease, presented with a fever and cough but no symptoms of hemoptysis, fatigue, or syncope within the previous 24 days. The chest computed tomography (CT) at approximately 2 weeks of the disease revealed wedge-shaped consolidations with pleural-based, hilar-pointing configurations (Figure 1). Outpatient antibiotic therapy (azithromycin and erythromycin) for 10 days, followed by inpatient dual broad-spectrum antibiotic therapy (minocycline and meropenem) for 13 days and immunosuppressive therapy [methylprednisolone (12 days) and intravenous immunoglobulin (3 days)] were initiated at the other hospital. However, the patient still presented with a persistent fever, persistent paroxysmal coughing, and wheezing. Thus, he was transferred to our hospital on 30 November 2023. At admission, *Mycoplasma pneumoniae*-polymerase chain reaction (MP-PCR) was positive, accompanied by elevated specific IgM of 48.8 (<20) and IgG >300. Moreover, the laboratory findings were white blood cell (WBC) level of 22.76 × 10^9 ^/L, neutrophil ratio (NEUT%) of 74.2% (the neutrophil count was 13.7 × 10^9 ^/L), procalcitonin (PCT) level of 0.83 ng/ml, and C-reactive protein (CPR) and serum amyloid A (SAA) levels of 75.05 and 232.3 mg/L, respectively. Moreover, the coagulation analysis revealed an elevated D-dimer level (5.18 mg/L) and elevated fibrin degradation products (FDP) (10.35 mg/L). Therefore, considering a double bacterial infection, he was intravenously administered linezolid (30 mg/kg/day, q8h) combined with oral moxifloxacin (10 mg/kg/day) as an anti-infection therapy, along with methylprednisolone (2 mg/kg/day, q12h) as an anti-inflammatory treatment and low-molecular-weight heparin (LMWH, 3,000 IU/day, qd) as an anticoagulation therapy (Table 1). Within the first 4 days after admission, his fever had gone, but the cough was still persistent. In addition, microbiological testing of sputum, bronchoalveolar lavage fluid, and blood samples for bacteria, fungi, and common respiratory viruses (including respiratory syncytial virus, adenovirus, influenza virus, rhinovirus, and human metapneumovirus) yielded negative results. On day 5, chest contrast-enhanced CT showed multiple filling defects in the bilateral lower lobe pulmonary arteries, involving the initial segments of some basal segmental arteries and their distal branches (Figure 2). Based on the above finding, bilateral PE with bilateral lower lung infarction was suspected. Thus, his dosage of low-molecular-weight heparin was adjusted to 6,000 IU/day, administered every 12 h. However, remarkably, coagulation examinations revealed a decrease in D-dimer level (0.97 mg/L) and fibrinogen (FIB) (4.35 g/L). On day 7, Gram-positive (G^+^) bacteria were found in sputum culture. After 15 days of hospitalization, the condition of the patient was stable. In addition, the posteroanterior and lateral chest radiographs indicated that the inflammation in the lungs had been partially reduced compared to before. The anticoagulation regimen was transitioned from low-molecular-weight heparin calcium to oral rivaroxaban (15 mg/day). He was discharged after an 18-day stay, with normalized inflammatory markers and clinical improvement. His oral rivaroxaban (15 mg/day) treatment was continued for 3 months. At the 3-month follow-up, he was afebrile with no cough or wheezing. Contrast-enhanced chest CT showed no evidence of thromboembolism in the bilateral pulmonary arteries or veins, but revealed residual sequelae of right lower lobe atelectasis with minimal necrosis (Figure 3).

**Table 1:** The basic information of the pediatric patient with SMPP.

Basic patient presentation	Case
Age (year)	9 years old
Gender	Boy
Embolism position	Bilateral pulmonary arteries
Fiberoptic bronchoscopy	Yes (hospital day 2 and 7)
Duration of hospitalization	31 days
Days of fever	28 days (intermittent)
Days of thrombus from onset	Hospital day 5
Days of thrombus disappear	3 months after leaving the hospital
Antibiotics before admission	(1) Azithromycin for 3 days and erythromycin for 6 days; (2) minocycline for 10 days and meropenem for 13 days
Antibiotics after admission	Linezolid and moxifloxacin
Anti-inflammatory therapy	Methylprednisolone
Anticoagulant therapy	Low-molecular-weight heparin and rivaroxaban

## 3 Discussion

In 2023, the post-COVID-19 era began, coinciding with a significant surge in MP infections, which reached epidemic levels globally (7). Furthermore, there has been a significant surge in reports of RMPP or severe MPP (SMPP) worldwide, with a particularly notable increase observed in Asia (8, 9). According to the *Guidelines for the Management of Community-acquired Pneumonia in Children* (2024 revision), RMPP is defined by the following criteria: (1) failure to respond to standard macrolide therapy for at least 1 week, (2) persistent fever, (3) progressive worsening of clinical symptoms, (4) deterioration of pulmonary imaging findings, and (5) presence of extrapulmonary complications involving multiple organ systems (10). RMPP mostly affects school-aged children (11). This child met the diagnostic criteria for both SMPP and RMPP, with the multifactorial pathogenesis attributable to a drug-resistant MP infection, hyperinflammatory response, and concurrent mixed infections. A delayed diagnosis likely contributed to disease progression, further complicating the clinical course. Moreover, the patient had residual sequelae of right lower lobe atelectasis. Early recognition and aggressive management of SMPP and RMPP are critical to minimizing the development of long-term sequelae (12). The optimal therapeutic window lies within 5–10 days post-fever onset (13). If the fever persists beyond 14 days of disease progression with no clinical improvement, patients are at high risk of irreversible complications (10). Given the clinical heterogeneity of MPP, individualized therapeutic regimens should be formulated based on disease subtypes (14, 15). Especially for SMPP cases, targeted multimodal interventions (e.g., combined anti-infective therapy, glucocorticoids, bronchoscopy, and anticoagulation) should be prioritized (16). It is also important to note that management must address both co-infections and precisely identify/control excessive inflammatory responses, including cytokine storms. Failure to promptly control hyperinflammation may significantly increase the risk of secondary infections and long-term sequelae (17).

MP infections can give rise to both pulmonary and extrapulmonary complications, such as necrotizing pneumonia (NP), plastic bronchitis (PB), thromboembolism, myocarditis, hemolytic anemia, Stevens–Johnson syndrome, and erythema multiforme (18–22). Significantly, there has been a rising number of reported cases of MPP-associated thromboembolism (23, 24). The potential mechanisms underlying MP-associated thromboembolism include the following (25). First, MP can directly invade and damage pulmonary vascular endothelial cells, leading to endothelial activation and exposure of tissue factor, thus initiating the extrinsic coagulation cascade and promoting localized thrombus formation. Second, the MP infection triggers a robust inflammatory response characterized by elevated cytokines, thus inducing vascular wall inflammation and thrombotic vascular occlusion through endothelial dysfunction. Finally, through molecular mimicry, MP triggers autoimmune responses characterized by autoantibody production (antiphospholipid antibodies and antiprothrombin antibodies) and complement system activation, thereby elevating coagulation factor levels and establishing a systemic hypercoagulable state that predisposes the patient to thrombus formation. Thromboembolic events associated with MP infection can occur in any anatomical site, including PE, deep vein thrombosis (DVT) of the lower extremities, intracardiac thrombosis, aortic thrombosis, cerebral infarction, cerebral venous sinus thrombosis (CVST), pulmonary venous thrombosis, renal vein thrombosis, and splenic infarction (26–29). PE is the most common manifestation and serves as a major contributor to pulmonary necrosis, residual atelectasis, and organizing pneumonia (6). In this pediatric case, the patient developed a pulmonary embolism, pulmonary infarction, and residual right lower lobe atelectasis with focal necrosis during the disease course. The trigger for these complications may be a prolonged and excessive inflammatory response. The pathogenesis of this dysregulated hyperinflammation likely stems from multiple factors: delayed initiation and inadequate dosing of corticosteroid therapy, macrolide antibiotic resistance, and concurrent bacterial co-infection. Therefore, the early recognition of and prophylactic intervention for a PE hold significant clinical relevance, particularly in mitigating life-threatening complications and improving long-term outcomes.

Chest pain represents the most frequently reported symptom of a PE in children with an MP infection (6, 30). While dyspnea and hemoptysis may also occur, these manifestations are often overshadowed by the overlapping symptoms of MPP, complicating their identification. Notably, one-seventh to one-third of pediatric PE cases present asymptomatically (20, 31). Beyond clinical evaluation, D-dimer levels provide a valuable diagnostic clue for a PE in MP infections (32). Despite its high sensitivity for detecting a PE, the D-dimer level lacks specificity, as it can also be markedly elevated in patients with SMPP without thrombotic events (3, 33, 34). In addition, the most common diagnostic method for MPP combined with a PE is chest contrast-enhanced CT (35). The direct imaging signs of a PE on CT include filling defects or complete obstruction within the vascular lumen (36). Other associated findings may involve a localized reduction in pulmonary vascular markings, patchy lung shadows, and dilated bronchial arteries (37). A PE can often lead to secondary pulmonary infarction, with its classic imaging manifestation being a wedge-shaped consolidation beneath the pleura, with the apex pointing toward the hilum (38). However, some patients may also exhibit a reversed halo sign on imaging (39). These signs are critical for guiding the early diagnosis of a pulmonary embolism. Throughout the clinical course, this child did not exhibit typical symptoms of a PE. However, his D-dimer levels exceeded 5 mg/L by week 4 of illness, and contrast-enhanced chest CT at week 5 confirmed a PE with pulmonary infarction. Notably, wedge-shaped consolidations with pleural-based, hilar-pointing configurations were already evident in the bilateral lower lobes on CT imaging in approximately week 2 of the disease. For such cases, a PE should be suspected early despite atypical presentations to mitigate thromboembolic complications and long-term sequelae. For SMPP complicated by a PE, anticoagulation is the cornerstone of therapy. Systemic thrombolysis should be considered if hemodynamic instability develops. Unfractionated heparin or LMWH is mostly used as an initial anticoagulation therapy. Moreover, for sequential anticoagulation, LMWH, warfarin, or rivaroxaban are generally selected (39). Anticoagulation therapy is typically continued for 3–6 months (40). In this case, the child received LMWH for 15 days during hospitalization, followed by oral rivaroxaban for 3 months, resulting in the resolution of the PE and favorable clinical outcomes. A phase 3 clinical trial evaluating rivaroxaban for pediatric acute venous thromboembolism reported that, compared to standard anticoagulants (LMWH or vitamin K antagonists), rivaroxaban reduced the risk of thrombotic recurrence without increasing bleeding risk (41). To prevent an MP-associated PE, the following strategies are recommended (10). First, early recognition and timely treatment of the MP infection to reduce progression to SMPP is recommended, thereby minimizing thrombotic complications. In addition, measures should be taken to minimize the risk of thrombosis, including ensuring adequate fluid intake to prevent dehydration caused by persistent high fever and avoiding prolonged immobility, among other precautions. Finally, it is necessary to monitor D-dimer levels in children with SMPP who have prolonged fever, extensive pulmonary consolidation, or elevated inflammatory markers. Clinicians should initiate prophylactic anticoagulation if the patient’s D-dimer level is elevated and there is no bleeding risk. Continuous assessment of bleeding risk during anticoagulation therapy is also important.

CT technology can only detect differences in tissue density, whereas magnetic resonance imaging (MRI) technology offers superior tissue contrast, multiplanar imaging capabilities, sensitivity to blood flow, and absence of ionizing radiation. These features make MRI particularly suitable for detecting and diagnosing soft tissue lesions in the chest, especially for radiation-sensitive populations (e.g., children). Chest soft tissues include the heart, mediastinum, pleura, and chest wall. However, MRI has limited utility in evaluating pulmonary diseases due to motion artifacts caused by physiological respiratory movements, low signal intensity from air-filled lungs, and magnetic field inhomogeneities at air/soft tissue interfaces. Currently, some noble gas MRI contrast agents, such as hyperpolarized (HP) butane gas (42), HP diethyl ether (DE) gas (43), parahydrogen-hyperpolarized propane-d_6_ gas (44), are revolutionizing functional pulmonary MRI. These agents enable high-resolution lung imaging and are compatible with virtually any MRI system, including emerging portable bedside low-field MRI systems.

## 4 Conclusion

This case highlights the critical risk of a PE in pediatric RMPP, even without classic thrombotic symptoms. Early recognition of subtle clinical clues, such as persistent fever unresponsive to conventional therapy and elevated inflammatory and coagulation markers (e.g., D-dimer), combined with contrasted chest CT, is essential for timely diagnosis. The successful outcome in this patient was achieved through a multidisciplinary approach integrating targeted anti-infective therapy, adjusted anticoagulation (escalated low-molecular-weight heparin followed by rivaroxaban), and immunomodulation. The complete resolution of the PE on follow-up imaging supports the efficacy and safety of a 3-month oral anticoagulation regimen in children. However, the residual pulmonary necrosis observed in this case emphasizes the importance of long-term monitoring for chronic sequelae. Proactive coagulation screening and early imaging in refractory cases are warranted. Further studies are essential to optimize pediatric-specific anticoagulation protocols and risk stratification.

## Data Availability

The original contributions presented in the study are included in the article/Supplementary Material, further inquiries can be directed to the corresponding author.
